# From sepsis to necrotizing enterocolitis with appendiceal perforation: a case report of successful salvage therapy with aztreonam–avibactam in an extremely preterm infant with NDM-producing *Klebsiella pneumoniae*

**DOI:** 10.3389/fphar.2026.1919037

**Published:** 2026-10-19

**Authors:** Yang He, Jichong Huang, Bin Du, Zhe Chen, Liang Huang, Jing Shi, Min Zhang, Li Zhang, Jun Tang

**Affiliations:** 1 Department of Pediatrics, West China Second University Hospital, Sichuan University, Chengdu, China; 2 Key Laboratory of Birth Defects and Related Diseases of Women and Children (Sichuan University), Ministry of Education, Chengdu, China; 3 Department of Neurology and State Key Laboratory of Biotherapy, National Clinical Research Center for Geriatrics, West China Hospital, Sichuan University, Chengdu, China; 4 Department of Pharmacy/Evidence-Based Pharmacy Center, West China Second University Hospital, Sichuan University, Chengdu, China; 5 Department of Pediatric General Surgery, West China Second University Hospital, Sichuan University, Chengdu, China

**Keywords:** aztreonam–avibactam, carbapenem-resistant *Klebsiella pneumoniae*, case report, extremely preterm infant, salvage therapy

## Abstract

Infections caused by New Delhi metallo-β-lactamase (NDM)-producing *Klebsiella pneumoniae* represent a major therapeutic challenge in neonatology, particularly in extremely preterm infants, for whom effective options are limited and dosing and safety data for last-resort agents such as aztreonam-avibactam remain scarce. We report the case of a male infants born at 24^+2^ weeks of gestation with a birth weight of 530 g who developed *Klebsiella pneumoniae* sepsis with poor clinical response to aztreonam combined with polymyxin B. His clinical course was complicated by pulmonary hemorrhage, disseminated intravascular coagulation, necrotizing enterocolitis, appendiceal perforation, and suspected necrotizing fasciitis. Blood and ascitic fluid metagenomic next-generation sequencing (mNGS) identified NDM-producing carbapenem-resistant *K. pneumoniae*. Given the lack of effective alternatives and ongoing clinical deterioration, off-label intravenous aztreonam-avibactam was initiated as salvage therapy. Marked clinical improvement was observed within 4 days after aztreonam-avibactam treatment, blood mNGS converted to negative by day 6, and skin and gastrointestinal manifestations resolved after 10 days of treatment, and no adverse events considered attributable to aztreonam-avibactam were identified. This case suggests that aztreonam-avibactam may be a viable salvage option for life-threatening NDM-producing *K. pneumoniae* infections in extremely preterm infants, though prospective pharmacokinetic and safety data in this population are urgently warranted.

## 1 Introduction

Infections caused by New Delhi metallo-β-lactamase (NDM)-producing *Klebsiella pneumoniae* represent a major therapeutic challenge in neonatology, particularly among extremely preterm infants. These pathogens exhibit resistance to nearly all β-lactam antibiotics, including carbapenems, thereby severely limiting available treatment options. Clinical management is further complicated by the extreme vulnerability of this population and the lack of robust safety and pharmacokinetic data for many last-resort antimicrobial agents.

Aztreonam–avibactam combines a monobactam that is stable against metallo-β-lactamases with a β-lactamase inhibitor capable of inhibiting co-produced serine β-lactamases. This complementary mechanism provides a strong pharmacological rationale for the treatment of infections caused by NDM-producing organisms. However, evidence supporting the efficacy and safety of aztreonam–avibactam in extremely low birth weight infants remains exceedingly limited.

To date, reports describing the use of aztreonam-avibactam for severe NDM-producing *K. pneumoniae* infection in an extremely preterm infant are lacking. This case provides rare real-world evidence regarding the potential role of aztreonam-avibactam as salvage therapy in one of the most fragile patient populations in clinical practice.

## 2 Case presentation

A male infant was born at 24^+2^ weeks of gestation with a birth weight of 530 g. The maternal history was notable for bilateral hydronephrosis, obesity, and an adverse obstetric history. Prenatal screening for *Ureaplasma urealyticum* and group B *Streptococcus* was negative, whereas vaginal secretion testing identified *Streptococcus anginosus*. Following initial resuscitation at birth, the infant was admitted to the neonatal intensive care unit and received invasive conventional mechanical ventilation. Empirical antimicrobial therapy was initiated with ampicillin and ceftazidime, following an initial episode of *Escherichia coli* bacteremia identified on blood culture. Following treatment, inflammatory markers normalized, antibiotics were discontinued at post-natal 14, and enteral feeding was gradually advanced.

On day 41 of life, when enteral feeding was advanced to 60 mL/kg/day, the infant developed pulmonary hemorrhage and disseminated intravascular coagulation, accompanied by scattered ecchymoses. Complete blood count revealed pancytopenia, with a nadir white blood cell count of 2.5 × 10^9^/L, platelet count of 29 × 10^9^/L, and hemoglobin level of 89 g/L. The C-reactive protein level was <0.5 mg/L. Metagenomic next-generation sequencing (mNGS) of blood detected *Klebsiella pneumoniae* (10 reads). Concurrent rectal swab screening was positive for carbapenem-resistant *K. pneumoniae* (CRKP). Antimicrobial therapy was therefore adjusted to polymyxin B, administered with a loading dose of 25,000 IU/kg (equivalent to 2.5 mg/kg) followed by 15,000 IU/kg (1.5 mg/kg) every 12 h (Tsuji et al., 2019), in combination with aztreonam at 30 mg/kg every 8 h. Although the procalcitonin level decreased to a nadir of 0.25 ng/mL, the pancytopenia did not improve substantially.

On day 56 of life, the infant weighed 1.41 kg. He developed progressive abdominal distension, gastric retention, accompanied by fever up to 37.7 °C. Physical examination revealed the abdomen to be markedly distended, with increased wall tension, tenderness, and absent bowel sounds. Fresh blood was noted on digital rectal examination. Laboratory tests showed leukopenia, with a white blood cell count of 1.2 × 10^9^/L, together with elevated inflammatory markers, including a C-reactive protein level of 31.8 mg/L and a procalcitonin level of 7.13 ng/mL. Imaging demonstrated ascites and pneumatosis intestinalis. Exploratory laparotomy confirmed neonatal necrotizing enterocolitis complicated by appendiceal perforation. Partial resection of necrotic bowel and appendectomy were performed, followed by terminal ileostomy. Postoperatively, blood mNGS detected *K. pneumoniae* with high confidence (3,087 species-specific sequence reads), resistance-gene reads were not separately reported for the blood specimen. Ascitic fluid mNGS detected *K. pneumoniae* with high confidence (259,429 species-specific sequence reads) and concurrently identified multiple antimicrobial resistance genes, including the NDM β-lactamase gene (101 reads), SHV β-lactamase gene (40 reads), APH (3′)-Ia (3 reads), and tet(A) (3 reads). *Enterococcus faecium* was also detected in ascitic fluid with moderate confidence, with 9 species-specific reads. Both blood and ascitic fluid cultures were negative. Vancomycin was administered at 15 mg/kg every 8 h, with a trough concentration of 19.3 μg/mL, in combination with polymyxin B and aztreonam. Given the high risk of thrombosis and microvascular ischemia associated with neonatal sepsis, disseminated intravascular coagulation, and extensive bowel necrosis, thrombin-antithrombin complex (TAT) was monitored serially after surgery using a protocol previously validated at our institution for critically ill neonates with infectious peritonitis (Zhang et al., 2024; Wang et al., 2025). In view of the infant’s thrombocytopenia and recent laparotomy, low-molecular-weight heparin (0.75 mg/kg subcutaneously every 12 h) was initiated for thromboprophylaxis in the presence of active thrombin generation, as TAT exceeded the institutional cutoff (>4 ng/mL). Anti-Xa activity was monitored at 0.2 IU/mL. Platelet counts, coagulation profiles, and cranial ultrasound were closely monitored throughout the treatment course.

On day 60 of life, the infant developed erythema, swelling, and progressive dark discoloration over the anterior chest, abdominal wall, and right lower limb (Figures 1A,D). The right leg became indurated and tense, although the dorsalis pedis artery remained palpable. The C-reactive protein level was 13.3 mg/L while platelet count remained persistently low. These findings were considered consistent with sepsis-associated cutaneous involvement, raising concern for progression to suspected necrotizing fasciitis. In the absence of surgical confirmation and given that the skin lesions resolved with antibiotic therapy alone, necrotizing fasciitis remains only a suspected diagnosis. Post-operative wound edema and transient abdominal compartment syndrome could not be completely excluded, however, such conditions rarely present as unilateral limb swelling. Based on the known rectal CRKP colonization, mNGS detection of the NDM-producing *K. pneumoniae* in ascitic fluid, and ongoing clinical deterioration despite treatment with polymyxin B combined with aztreonam, an off-label antimicrobial regimen was adopted after full risk-benefit evaluation by multidisciplinary clinicians and the patient’s legal guardian. Antimicrobial therapy was switched to aztreonam-avibactam, dosed 40 mg/kg (aztreonam component at 30 mg/kg and avibactam component at 10 mg/kg) every 8 h, with each dose infused over 3 h. Linezolid was also added at 10 mg/kg every 8 h.

**FIGURE 1:**
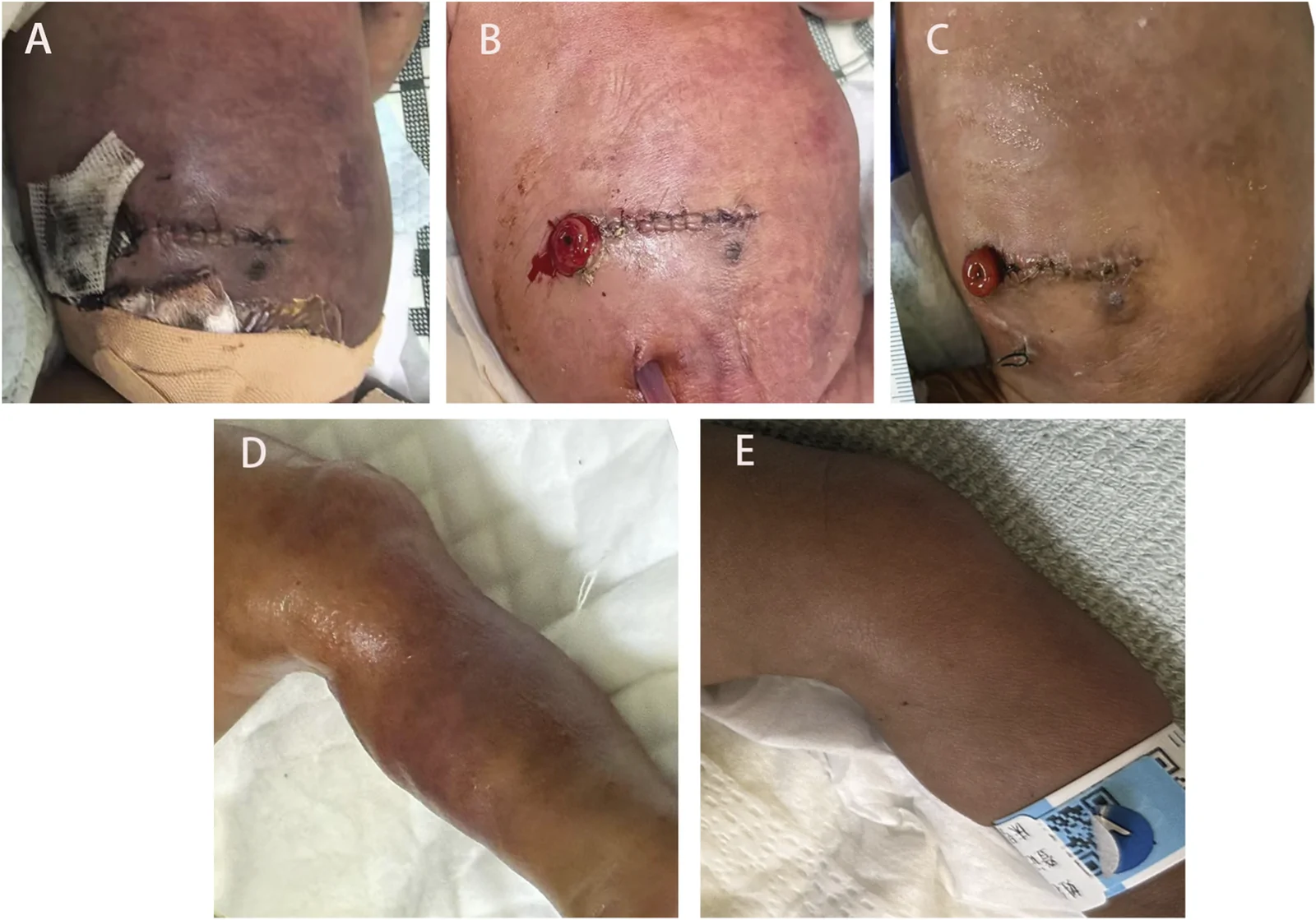
Clinical photographs of the infant. **(A,D)** Day 60, ecchymoses on the lower legs and abdomen. **(B)** Day 64, improved purpuric discoloration over the abdomen. **(C,E)** Day 70, resolution of ecchymoses on the lower legs and abdomen.

On day 64 of life (the fourth day of aztreonam-avibactam therapy), the infant showed marked clinical improvement, with substantial resolution of ecchymoses and abdominal distension, recovery of bowel sounds (Figure 1B), and a decline in C-reactive protein to 3.7 mg/L.

On day 66 of life (the sixth day of aztreonam-avibactam therapy), blood mNGS became negative, linezolid was discontinued, and enteral feeding was restarted.

On day 70 of life (the 10th day of aztreonam-avibactam therapy), the ecchymoses and induration of the right lower limb had completely resolved (Figures 1C,E), enteral nutrition was advanced smoothly, and the C-reactive protein level further decreased to 0.6 mg/L.

On day 84 of life (the twenty-fourth day of aztreonam-avibactam therapy), respiratory support was transitioned to non-invasive ventilation. Given the complicated postoperative intra-abdominal infection, aztreonam-avibactam was continued for approximately 14 days after the obvious clinical response on day 70 (the 10th day of aztreonam-avibactam therapy), completing a total 24-day therapy (Days 60–84). No dose adjustment was required during the therapy due to no hepatic or renal impairment. The full timeline and laboratory results were shown in Table 1 and Figure 2.

**TABLE 1:** Summary of key laboratory findings, antibiotic administration and clinical events.

Day of life	Complete blood count			Inflammatory markers		Hepatic function				Renal function		Antibiotics	Clinical events
	WBC (×10^9^/L)	Hb (g/L)	PLT (×10^9^/L)	CRP (mg/L)	PCT (ng/mL)	ALT (U/L)	AST (U/L)	ALB (g/L)	DBIL (μmol/L)	Cr (μmol/L)	BUN (mmol/L)		
D41	2.5	95	40	<0.5	0.51	—	—	—	—	—	—	Polymyxin B Aztreonam	PH/DIC; pancytopenia
D41	3.5	125	29	<0.5	—	—	—	—	—	—	—		
D43	4.1	111	86	<0.5	—	9	20	24.4	12.7	2.6	23		Blood mNGS: *Klebsiella pneumoniae*
D50	3.9	100	68	<0.5	0.29	9	24	30.7	10.7	3	41		
D55	3.5	123	114	7.5	6.32	—	—	—	—	—	—		
D56	1.2	118	97	31.8	7.13	9	27	28.5	17.7	4.6	31	Polymyxin B Aztreonam Vancomycin	NEC with appendiceal perforation; surgery Blood mNGS: *Klebsiella pneumoniae* Ascitic fluid mNGS: CRKP + *Enterococcus faecium*
D56 (Post-operavtion)	2.1	100	84	52.2	—	—	—	—	—	—	—		
D57	3.3	97	96	72.9	8.36	9	32	34.9	30.1	7.7	34		
D60 (D0*)	4.5	97	43	13.3	—	—	—	—	—	—	—	ATM-AVI Linezolid	Suspected necrotizing fasciitis
D64 (D4*)	4.6	103	44	3.7	—	—	—	—	—	—	—		Clinical improvement
D66 (D6*)	5.3	106	65	2.5	—	9	28	39.1	26.2	7.6	27	ATM-AVI	Blood mNGS: Negative; linezolid stopped
D70 (D10*)	4.7	89	63	0.6	—	—	—	—	—	—	—		Skin lesions resolved
D73	5.2	89	87	<0.5	—	9	26	39.8	16.9	8	19		
D82	6.9	103	153	<0.5	—	28	38	43.2	17.4	—	—		
D84 (D24*)	7.8	88	185	<0.5	0.12	—	—	—	—	—	—	Discontinued	ATM-AVI discontinued; NIV transition

Footnotes: D0*-D24* indicate the day of ATM-AVI therapy (initiated on Day 60). PH/DIC: pulmonary hemorrhage/disseminated intravascular coagulation; NEC: necrotizing enterocolitis; mNGS: metagenomic next-generation sequencing; ATM-AVI: aztreonam-avibactam; NIV: non-invasive ventilation; CBC: complete blood count; Hb: hemoglobin; PLT: platelet count; CRP: C-reactive protein; PCT: procalcitonin; ALT: alanine transaminase; AST: aspartate transaminase; DBIL: direct bilirubin; Cr: creatinine; BUN: blood urea nitrogen; CRKP: carbapenem-resistant *Klebsiella pneumoniae*; CRP <0.5 mg/L is reported as below the detection limit. '—' indicates the parameter was not measured on that day.

**FIGURE 2:**
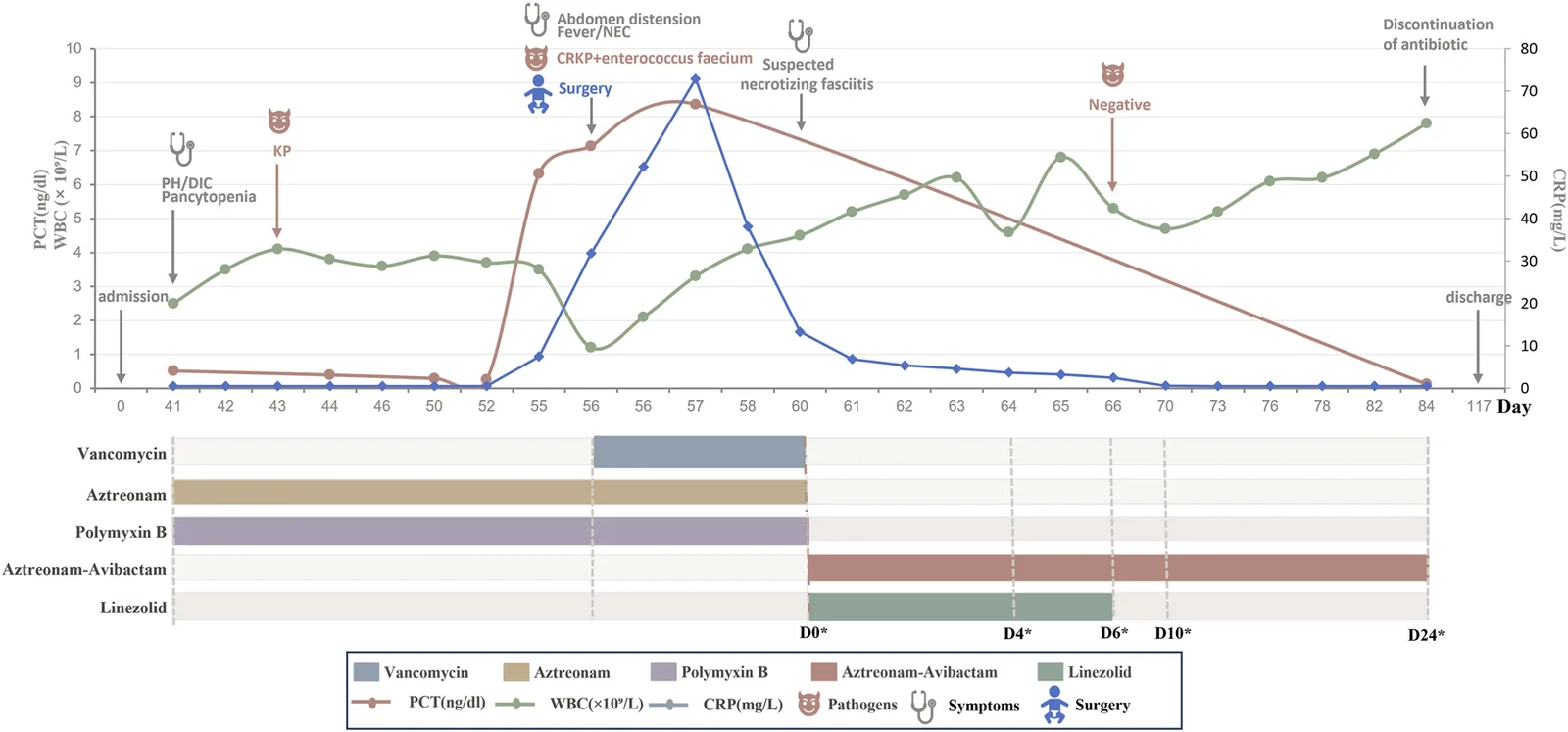
Clinical timeline of the patient.

On day 89 of life, corresponding to postoperative day 33, the infant had achieved full enteral feeding with stable weight gain. He was discharged after 117 days of hospitalization, with a discharge weight of 2,320 g.

## 3 Discussion

Necrotizing enterocolitis (NEC) is one of the most severe gastrointestinal emergencies in the neonatal period and remains a leading cause of mortality, particularly among preterm infants. Its incidence is strongly associated with gestational age and birth weight, with the highest risk observed in extremely preterm and extremely low birth weight infants (He et al., 2024). A large meta-analysis involving 574,692 neonates reported a pooled global incidence of NEC of 6.0% (95% confidence interval 4.0%–9.0%) in very low birth weight populations (Alsaied et al., 2020). Disruption of the intestinal barrier, bacterial translocation, and sepsis-associated multi-organ dysfunction are key drivers of mortality in affected infants. The most commonly implicated pathogens include *Klebsiella pneumoniae*, *Enterococcus* species, and *Clostridium* species (Wang et al., 2024; Schönherr-Hellec and Aires, 2019).

Among these, carbapenem-resistant *Klebsiella pneumoniae* (CRKP) has emerged as a particularly concerning pathogen in hospitalized neonates, given its limited treatment options and high associated mortality. A systematic review including 128 studies reported a pooled prevalence of CRKP infection of 0.3% in hospitalized neonates, with a mortality rate of 22.9% (95% CI: 13.0%–32.9%) (Hu et al., 2023). Notably, molecular epidemiological analyses indicate that approximately 75.3% of CRKP isolates harbor metallo-β-lactamases (MBLs), among which New Delhi metallo-β-lactamase (NDM) is the predominant subtype, accounting for 64.3% of cases (Zhou et al., 2024).

NDM enzymes confer resistance to nearly all β-lactam antibiotics, including carbapenems, thereby severely limiting therapeutic options. However, aztreonam retains intrinsic stability against hydrolysis by MBLs, although it is susceptible to co-produced serine β-lactamases. Avibactam, a non-β-lactam β-lactamase inhibitor, effectively inhibits class A, C, and some class D β-lactamases, thereby protecting aztreonam from degradation. This complementary mechanism provides a strong pharmacological rationale for the use of aztreonam-avibactam in the treatment of infections caused by NDM-producing organisms. Accordingly, several recent expert recommendations and clinical guidelines have supported this combination as a preferred therapeutic option for infections due to MBL-producing Enterobacterales.

Aztreonam-avibactam was approved in China on 30 June 2025 for the treatment of complicated intra-abdominal infections and hospital-acquired pneumonia, including ventilator-associated pneumonia, in adults with limited or no alternative therapeutic options. However, no approved indication currently exists for neonatal populations. In the present case, the causative organism was an NDM-producing *Klebsiella pneumoniae*, which exhibits intrinsic resistance to carbapenems. Given the lack of clinical response to prior therapy with polymyxin B plus aztreonam, further therapeutic escalation was imperative, as treatment options were otherwise extremely limited and the risk of mortality was high.

Although direct clinical evidence supporting the use of aztreonam-avibactam in neonates remains scarce, the underlying therapeutic strategy, protection of aztreonam from NDM-mediated hydrolysis by avibactam, has been indirectly supported in neonatal settings. Prior to this case, our center successfully treated another extremely preterm infant with invasive infection caused by a metallo-β-lactamase-producing CRKP using a combination regimen of ceftazidime-avibactam plus aztreonam. In addition, a retrospective study including six neonates, of whom two were infected with NDM-producing organisms, reported survival in all cases without observed adverse events following treatment with ceftazidime-avibactam combined with aztreonam (Guang et al., 2025).

Importantly, the fixed combination of aztreonam-avibactam offers practical and pharmacological advantages over combination regimens. It simplifies administration, reduces the risk of dosing mismatch between agents, and ensures pharmacokinetic synchrony of both components, which may be particularly relevant in critically ill neonates with highly variable drug distribution and clearance.

A phase IIa pediatric clinical trial evaluating aztreonam-avibactam in neonates and infants younger than 9 months (NCT06462235) was initiated in 2024 and is expected to provide critical pharmacokinetic data to inform dosing strategies and safety evaluation in this vulnerable population.

This case also highlights the diagnostic challenges of severe infections in high-risk neonatal populations. The diagnostic yield of conventional microbiological cultures is often limited by small blood sample volumes, prior antibiotic exposure, and low pathogen burden, especially in immunocompromised patients or those undergoing repeated invasive procedures (Luo et al., 2021). In contrast, mNGS, with its high sensitivity and broad pathogen detection capability, enables the identification of causative organisms and antimicrobial resistance determinants even when conventional cultures remain negative (Balks et al., 2024). In the present case, the concordant detection of *K. pneumoniae* in both blood and ascitic fluid mNGS while cultures were negative was consistent with our previous findings, supporting the reliability of this approach in critically ill neonates (Zhang et al., 2025). Therefore, in scenarios where infection is strongly suspected but conventional diagnostics are inconclusive, mNGS of blood and primary infection sites may serve as a valuable adjunctive tool to guide individualized antimicrobial therapy and potentially improve clinical outcomes.

In this case, informed consent was obtained from the patient’s guardians before initiation of off-label therapy, and treatment decisions were guided by a multidisciplinary team. Aztreonam-avibactam dosing was calculated based on the aztreonam component. Clinical improvement was evident within 4 days after aztreonam-avibactam treatment, as reflected by resolution of symptoms, improvement in physical findings, and normalization of inflammatory markers. By day 10, the clinical manifestations had resolved, and repeat blood mNGS became negative shortly after treatment initiation. Aztreonam-avibactam was initiated on day 60 and continued for a total of 24 days, given the complicated intra-abdominal infection with suspected necrotizing fasciitis in an extremely preterm infant. Therapy was continued for approximately 14 days after the obvious clinical response on day 70 (the 10th day of aztreonam-avibactam therapy), consistent with the principles of Chinese pediatric intra-abdominal infection guidelines for complicated infection. Throughout treatment, close monitoring for adverse events, including hepatic and renal function were performed, and no adverse events considered attributable to aztreonam-avibactam were identified.

In addition to *Klebsiella pneumoniae*, the ascitic fluid mNGS also detected *Enterococcus faecium* (9 reads, moderate confidence). Anti-Gram-positive therapy including vancomycin/linezolid was administered to cover Gram-positive pathogens during the period of clinical instability for this critical ill infant. We acknowledge that these agents may have contributed to the observed clinical improvement, and the favorable outcome cannot be attributed solely to aztreonam-avibactam.

Furthermore, effective surgical source control is a cornerstone for complicated intra-abdominal infection. Inflammatory markers kept rising pre-operatively. After surgical intervention combined with aztreonam-avibactam therapy, rapid clinical resolution and a marked decline in inflammatory parameters were observed. The good outcome was thus the result of combined surgical and antimicrobial management.

This case may represent one of the smallest and most premature infants successfully treated for NDM-producing *K. pneumoniae* infection with aztreonam-avibactam. It provides rare preliminary evidence supporting the potential effectiveness and tolerability of this regimen in an extremely vulnerable population. Nevertheless, these findings should be interpreted with caution because causality cannot be definitively established from a single case.

This study has several limitations. First, due to the absence of established neonatal dosing recommendations for aztreonam-avibactam, the dosing regimen in this case was determined through multidisciplinary consultation and extrapolated from standard neonatal aztreonam dosing. This approach may not fully achieve optimal pharmacokinetic and pharmacodynamic targets. In addition, a systematic pharmacokinetic assessment of aztreonam-avibactam was not performed, as therapeutic drug monitoring for this combination was unavailable at our institution. Second, conventional cultures were negative, and pathogen identification relied primarily on mNGS, although the concordant detection of *K. pneumoniae* in blood and ascitic fluid, together with compatible clinical manifestations, strongly supported its etiological role. We did not arrange standardized microbiological surveillance after treatment withdrawal to confirm permanent pathogen clearance although the blood mNGS was negative at day 6 of aztreonam-avibactam therapy. Third, linezolid was administered concomitantly during the early phase of salvage therapy, which could cover Gram-positive organisms, and complicated attribution of clinical improvement solely to aztreonam-avibactam. Last, no patient’s legal guardian perspective was collected for this retrospective case report, which represents a limitation of this work according to the CARE guidelines.

Further real-world studies and prospective clinical trials are urgently needed to define the dose-response relationship, pharmacokinetic and pharmacodynamic targets, efficacy, and safety profile of aztreonam-avibactam in neonates with carbapenem-resistant *K. pneumoniae* infections. This is particularly important for extremely preterm and extremely low birth weight infants, for whom effective antimicrobial agents with favorable safety profiles and minimal nephrotoxicity are urgently needed.

## 4 Conclusion

This case describes the successful management of an extremely preterm infant with life-threatening NDM-producing *K. pneumoniae* infection complicated by necrotizing enterocolitis, appendiceal perforation, and suspected necrotizing fasciitis, using combined surgical source control and off-label aztreonam-avibactam salvage therapy. The favorable clinical response and absence of observed drug-related adverse events suggest that aztreonam-avibactam may be a potential therapeutic option for severe NDM-producing Enterobacterales infections in critically ill neonates when alternative treatments are limited. However, further pharmacokinetic, safety, and prospective clinical data are required before this regimen can be recommended more broadly in neonatal populations.

## Data Availability

The original contributions presented in the study are included in the article/Supplementary Material, further inquiries can be directed to the corresponding authors.
